# Herbal medicine *Yukgunja-tang* for functional dyspepsia protocol for a systematic review of randomized controlled trials

**DOI:** 10.1097/MD.0000000000012555

**Published:** 2018-10-05

**Authors:** Seok-Jae Ko, Jae-Woo Park, Jae-hong Lee, Soo-ho Cho, Jaehyung Lee, Seonguk Nam, Jinsung Kim

**Affiliations:** Department of Gastroenterology, College of Korean Medicine, Kyung Hee University, Seoul, Republic of Korea.

**Keywords:** functional dyspepsia, randomized controlled trial, systematic review, *Yukgunja-tang*

## Abstract

**Background:**

Functional dyspepsia (FD) is a common functional gastrointestinal disorder characterized by recurrent dyspeptic symptoms. *Yukgunja-tang* (YGT) is a traditional herbal formula that has been used for treating FD. This systematic review protocol aims to provide a guideline for investigating the efficacy and safety of YGT in the treatment of FD.

**Methods:**

The following databases will be searched from their inception until July 2018: Medline (via PubMed), EMBASE, the Cochrane Central Register of Controlled Trials (CENTRAL), Allied and Complementary Medicine Database (AMED), KoreaMed, National Digital Science Library (NDSL), Korean Medical Database (KMbase), Oriental Medicine Advanced Searching Integrated System (OASIS), Korean Studies information Service System (KISS), China National Knowledge Infrastructure Database (CNKI), and Citation Information by Nii (CiNii). Randomized controlled trials (RCTs) that used YGT or modified YGT for treating FD will be included. The control groups in these RCTs will include placebo, no-treatment waiting, and conventional western medicine groups. Trials testing YGT as an adjunct to western medicine for synergistic effect will also be included. The main outcome will be the total clinical efficacy rate. Data extraction and risk of bias assessment will be performed by two independent assessors. RevMan V.5.3 will be used for data analysis.

**Results:**

This study will provide a high-quality synthesis of current evidence of YGT for FD from several aspects including dyspepsia-related symptoms, quality of life and adverse events.

**Conclusion:**

The conclusion of our systematic review will provide evidence to judge whether YGT is an effective intervention for patient with FD.

**Ethics and dissemination:**

The protocol does not need ethics approval because identifying information of the participants will not be revealed. The systematic review will be published in a peer-reviewed journal and disseminated electronically and in print.

**Trial registration number:**

PROSPERO CRD42018090139.

## 1 Introduction

Functional dyspepsia (FD) is a pathological condition characterized by chronic or recurrent gastrointestinal symptoms, such as abdominal fullness, epigastric pain, or early satiation without evidence of structural organic lesions upon evaluation by esophagogastroduodenoscopy.^[1]^ The global prevalence of FD ranges from 11.5% to 29.2%.^[2,3]^ The pathophysiological mechanisms underlying FD remain unclear.^[4,5]^ Currently, pharmacotherapies including antisecretory agents, prokinetics, and proton pump inhibitors are used to treat FD; however, due to the unsatisfactory results of conservative treatments, many patients are seeking alternative and complementary medicines such as herbal medicine.^[6]^

*Yukgunja-tang* (YGT), which is called *Liu Jun Zi Tang* in China and *Rikkunshito* in Kampo medicine, is a herbal medicine consisting of the following 8 crude drugs: *Atractylodes japonica*, *Pinellia ternata*, *Ziziphus jujuba*, *Zingiber officinale*, *Poria cocos*, *Citrus unshiu*, *Panax ginseng*, and *Glycyrrhiza uralensis*. YGT has been used for the treatment of upper gastrointestinal disorders such as FD in Asia.^[7]^ YGT has been known to facilitate emptying of the stomach leading to an improvement in gastric motility and dyspeptic symptoms.^[8,9]^ YGT has also been proven to improve acyl ghrelin release, which is associated with gastric motor activity and food intake.^[10]^ A recent systematic review and meta-analysis suggested that YGT could potentially manage dyspeptic symptoms, although the quality of included studies was poor.^[7,9]^ However, the reviews were focused on gastrointestinal dysfunction or anorexia rather than FD,^[9]^ included only Chinese studies,^[11]^ and did not conduct a comparison between YGT and YGT with western medicine.^[7]^ Results from previous reviews did not produce adequate evidence to support recommendations for FD. The treatment for FD still remains unidentified. This review will aim to systematically synthesize the primary studies that compare the efficacy and safety of YGT for treating FD to that of Western medicine or placebos. This study will also review the physiology and clinical benefits of YGT-western medicine combination therapies.

## 2 Methods and analysis

### 2.1 Inclusion criteria for study selection

#### 2.1.1 Types of studies

The protocol for this systematic review will include randomized controlled trials (RCTs) and quasi-RCTs. Animal studies, case reports, and commentaries will be excluded.

#### 2.1.2 Types of patients

Patients with FD diagnosed based on the ROME criteria will be included regardless of age, gender, and race in this systematic review. The ROME criteria were first announced in 1992 as a standard for diagnosing FD. In 2016, ROME IV criteria were finalized after several revisions. In the case of studies conducted before 1991 when there were no standard criteria for diagnosing FD, studies using criteria similar to the ROME criteria (e.g., persistent and recurrent dyspepsia without organic lesions) will be selected based on the consensus of 2 reviewers (JP and SK). Dyspepsia from secondary pathologies such as gastro-esophageal reflux diseases and irritable bowel syndrome will be excluded.

#### 2.1.3 Types of interventions

Randomized studies of YGT either sole or modified treatment that some herbs are added will be included. Studies comparing YGT with any type of control intervention will also be included. Control groups will receive a placebo YGT, which has the same color and odor as YGT, no treatment waiting group, and conventional western medicine such as prokinetics, antidepressants, and proton pump inhibitor. The intervention in this review will include YGT-western medicine combination therapy compared with western medicine only.

#### 2.1.4 Types of outcome measures

The total clinical efficacy rate will be the primary outcome measure. Secondary outcomes will be dyspepsia-related symptom score, Short-Form health survey as a quality of life scale, adverse events, Hamilton Depression Rating Scale, gastric emptying time, recurrence 6 months after treatment, Gastrointestinal Symptom Rating Scale, and acylated ghrelin levels.

### 2.2 Data sources

The following databases will be searched from inception to July 2018: Medline (via PubMed), EMBASE, the Cochrane Central Register of Controlled Trials, and Allied and Complementary Medicine Database. We will also search 5 Korean Medical Databases including KoreaMed, National Digital Science Library, Korean medical Database, Oriental Medicine Advanced Searching Integrated System, and Korean Studies Information Service System. We will also search other Asian databases such as China National Knowledge Infrastructure Database in Chinese and Citation Information by Nii in Japanese. Trial registries such as clinicaltrials.gov and Clinical Research Information Service will also be searched. The search term will consist of the disease term part (e.g., intestine, digestion, stomach, gut, dyspepsia, discomfort, disturbance, pain, and dysfunction) and the intervention term part (e.g., *Yukgunja-tang*, *Liu Jun Zi Tang*, *Rikkunshito*, herbal medicine, medicine, and botanical). The search strategies designed for Medline (via PubMed) are presented in the Table 1. Modified search strategies will be applied to the other databases. No language restriction will be imposed.

**Table 1:**
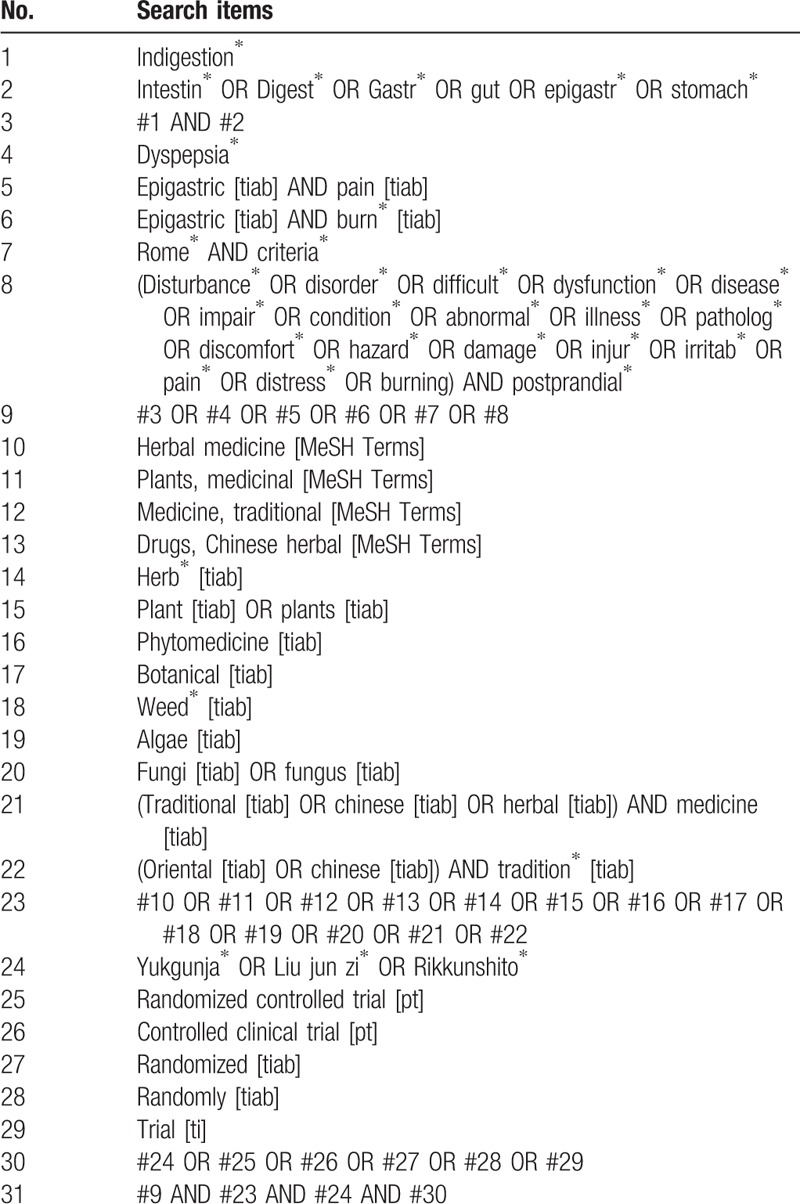
Search strategy used in PubMed.

### 2.3 Data collection and analysis

#### 2.3.1 Selection of studies

**Figure 1:**
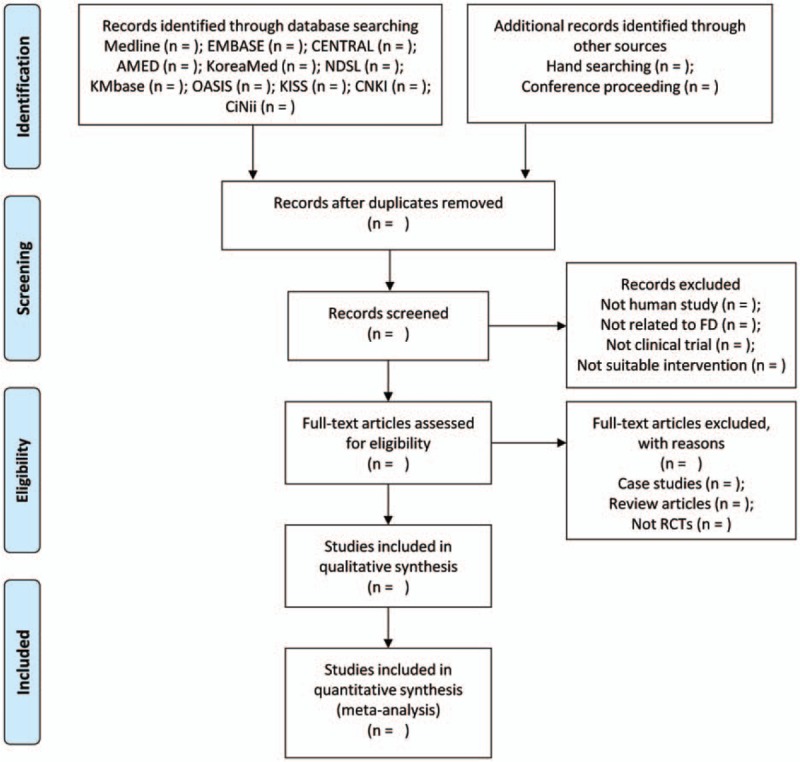
Flow chart of the search process.

Two reviewers (JL and SC) will independently review the titles, abstracts, and manuscript of the studies for eligibility for inclusion in the analysis. All reviewers will receive training for the process and purpose of selection. All studies identified by electronic and hand searches will be uploaded to Endnote X7 (Clarivate Analytics). The reasons for excluding studies will be recorded and shown in PRISMA-flow chart (Fig. 1). Any disagreement will be resolved based on a consensus and discussion between the 2 reviewers. If necessary, the arbiter (JP) will intervene and resolve the disagreement.

#### 2.3.2 Data extraction and management

Two review authors (JL and SC) will independently extract the data and fill out the standard data extraction form, which includes study information such as the first author, publication year, written language, research design, interventions, treatment period, outcome measures, main results, and statistics. Any discrepancies will be resolved by a discussion between the 2 reviewers, and if necessary, an arbiter (JP) will intervene to resolve the issue.

#### 2.3.3 Assessment of the risk of bias in the included studies

Two reviewers (JL and SC) will evaluate the risk of bias based on Cochrane Collaboration's Tool, which includes the following items: random sequence generation (selection bias), allocation concealment (selection bias), blinding of participants and personnel (performance bias), blinding of outcome assessment (detection bias), incomplete outcome data (attrition bias), selective reporting (reporting bias), and other bias. The result of the evaluation will be shown as one of 3 categories: low, unclear, and high. Any disagreement will be solved by discussion between the 2 reviewers, and if necessary, an arbiter (JP) will intervene.

#### 2.3.4 Measurement of treatment effect

We will use the mean difference with 95% CIs to assess continuous data, while relative risk with 95% CIs to evaluate dichotomous data.

#### 2.3.5 Unit of analysis issue

We will use only 1st-phase data in case of randomized cross-over trials to avoid carry-over effects. When the trials have multiple intervention groups, a pair-wise comparison will be made.

#### 2.3.6 Dealing with missing data

We will try to contact the original investigators by email if there are any missing or insufficient data. The statistical analysis will be performed base on intent-to-treat principle. If we cannot obtain missing data, it will be sought from the original source or trial reports.

#### 2.3.7 Assessment of heterogeneity

A random effects model will be used for the meta-analysis. We will test for heterogeneity using a χ^2^ test with a significance level of *P* < .1 and forest plot. We will also use *I*^2^ statistics, with *I*^2^ ≥ 50% indicating substantial heterogeneity. In case of heterogeneity, we will investigate the possible cause using a subgroup analysis or sensitivity analysis.

#### 2.3.8 Assessment of publication biases

In case the analysis includes more than 10 studies, a funnel plot will be generated to assess publication bias or small-study effects.

#### 2.3.9 Data synthesis

Statistical analyses will be performed by the Review Manager program (V.5.3 Copenhagen: The Nordic Cochrane Centre, The Cochrane Collaboration, 2014). Studies will be synthesized according to the type of intervention and/or control as follows: YGT versus conventional western medicine, YGT versus no treatment, YGT versus placebo control, and YGT combined by conventional western medicine versus only conventional western medicine. The YGT group will be permitted to include YGT with added as described in the “types of intervention” section.

#### 2.3.10 Subgroup analysis

A subgroup analysis will be conducted if there are sufficient subgroup studies to investigate the cause of heterogeneity. Subgroup analysis criteria will include the duration of treatment, prescription based on pattern identification according to Traditional Chinese Medicine, physical form of YGT such as granules or decoctions, and the number and species of added herbs. Low quality of studies can be removed to examine robustness of the results.

#### 2.3.11 Sensitivity analysis

The consolidated standards of reporting trials extension for herbal interventions will be used to assess the methodological and reporting quality of the studies. A sensitivity analysis will be performed to assess the robustness of the meta-analysis results.

#### 2.3.12 Grading the quality of evidence

Grading of Recommendations Assessment and Development and Evaluation will be used to investigate the quality of evidence. The quality of the level of evidence will be presented as one of 4 rankings, which are high, moderate, low, and very low.

## 3 Discussion

Several previous studies have investigated the effect of YGT on FD. One meta-analysis has shown that YGT and *Xiang Sha* YGT (YGT added to *Amomum villosum* and costus root) may be more effective than prokinetics for treating FD without side effects.^[7]^ Another recent systematic review showed that YGT had ameliorative effects on the adverse reactions induced by Western medicine and could achieve synergistic effects in FD patients.^[9]^ Evidence-based clinical practice guidelines for FD in Japan suggest using herbal medicine as a 2nd-line treatment (evidence level A). YGT was mentioned as an example of a herbal medicine.^[12]^ Based on previous studies, YGT has been considered a promising treatment for FD. The evidence for the efficacy of YGT in the treatment of FD and the underlying mechanism is continuously being updated.

This systematic review will reveal the available evidence for the efficacy of YGT and formulas composed of the addition of several herbs to YGT (such as *Xiang Sha* YGT) for the treatment of FD. We will also assess the efficacy of both YGT and combination therapy of YGT and western medicine and compare it with that of Western medicine alone or placebo. This review will provide an update on the latest RCTs of YGT and will be the first systematic review that focuses on the efficacy of YGT, herbs-added YGT, and YGT-Western medicine combination therapy on FD. We expect that this study will offer detailed information relevant to clinical practice and will be useful for the development of a Korean medicine guideline for FD.

One potential limitation that may influence the conclusions drawn from the study is the issue of pattern identification. In Traditional Chinese Medicine, FD can be divided into different syndromes based on the clinical symptoms and signs. In addition, most FD patients were found to have “spleen-deficiency and qi-stagnation syndromes.”^[13]^ Although YGT has been considered a herbal formula for invigorating the spleen and regulating qi,^[14]^ it may not be the best treatment for syndromes of FD other than “spleen-deficiency” and “qi-stagnation” syndromes.” Due to insufficient data on pattern identification in FD, we will include all studies that used YGT for the treatment of FD. Another expected limitation of this study will be poor quality reporting and methodology. In addition, most papers will be skewed toward the Chinese language. Nevertheless, this systematic review should further our understanding of herbal medicine for the treatment of FD.

This protocol for a review does not need any ethical approval because there is no primary data collection and only published data will be included. The systematic review will be published in a peer-reviewed journal and disseminated electronically or in print. Updates of the review will be performed and the results will be presented in international conferences.

## Author contributions

**Conceptualization:** Seok-Jae Ko.

**Data curation:** Seok-Jae Ko, Jae-Woo Park, Jae-hong Lee, Soo-ho Cho, Jaehyung Lee, Seonguk Nam, Jinsung Kim.

**Formal analysis:** Jae-Woo Park.

**Investigation:** Jae-hong Lee, Soo-ho Cho, Jaehyung Lee, Seonguk Nam.

**Methodology:** Jae-Woo Park, Jae-hong Lee, Soo-ho Cho, Jaehyung Lee, Seonguk Nam.

**Resources:** Jinsung Kim.

**Supervision:** Jae-Woo Park.

**Writing – original draft:** Seok-Jae Ko.

**Writing – review & editing:** Seok-Jae Ko.

## References

[1] Drossman DA The functional gastrointestinal disorders and the Rome III process. Gastroenterology 2006;130:1377–90. 16678553 10.1053/j.gastro.2006.03.008

[2] Shaib Y El-Serag HB The prevalence and risk factors of functional dyspepsia in a multiethnic population in the United States. Am J Gastroenterol 2004;99:2210–6. 15555004 10.1111/j.1572-0241.2004.40052.x

[3] Bernersen B Johnsen R Straume B Non-ulcer dyspepsia and peptic ulcer: the distribution in a population and their relation to risk factors. Gut 1996;38:822–5. 8984017 10.1136/gut.38.6.822 PMC1383186

[4] Aoki S Haruma K Kusunoki H Evaluation of gastric emptying measured with the 13C-octanoic acid breath test in patients with functional dyspepsia: comparison with ultrasonography. Scand J Gastroenterol 2002;37:662–6. 12126243 10.1080/00365520212508

[5] Talley NJ Phillips SF Non-ulcer dyspepsia: potential causes and pathophysiology. Ann Intern Med 1988;108:865–79. 3285748 10.7326/0003-4819-108-6-865

[6] Tominaga K Arakawa T Kampo medicines for gastrointestinal tract disorders: a review of basic science and clinical evidence and their future application. J Gastroenterol 2013;48:452–62. 23503839 10.1007/s00535-013-0788-z PMC3698434

[7] Xiao Y Liu YY Yu KQ Chinese herbal medicine liu jun zi tang and xiang sha liu jun zi tang for functional dyspepsia: meta-analysis of randomized controlled trials. Evid Based Complement Alternat Med 2012;2012:936459. 23304226 10.1155/2012/936459 PMC3530827

[8] Suzuki H Inadomi JM Hibi T Japanese herbal medicine in functional gastrointestinal disorders. Neurogastroenterol Motil 2009;21:688–96. 19563404 10.1111/j.1365-2982.2009.01290.x PMC2943019

[9] Mogami S Hattori T Beneficial effects of rikkunshito, a Japanese kampo medicine, on gastrointestinal dysfunction and anorexia in combination with Western drug: a systematic review. Evid Based Complement Alternat Med 2014;2014:519035. 24778703 10.1155/2014/519035 PMC3979068

[10] Kojima M Hosoda H Date Y Ghrelin is a growth-hormone-releasing acylated peptide from stomach. Nature 1999;402:656–60. 10604470 10.1038/45230

[11] Zeng G Chen G Mo X A meta-analysis of Xiangsha Liujunzi Decoction in treatment of functional dyspepsia. J Liaoning Univ TCM 2013;15:123–5.

[12] Miwa H Kusano M Arisawa T Japanese Society of Gastroenterology. Evidence-based clinical practice guidelines for functional dyspepsia. J Gastroenterol 2015;50:125–39. 25586651 10.1007/s00535-014-1022-3

[13] Zhang SS Chen Z Xu WJ Study on distribution characteristic of syndrome of 565 cases of functional dyspepsia by twice differentiation of symptoms and signs based on the “cold, heat, deficiency, excess”. Chin J Tradit Chin Med Pharm 2008;23:833–4.

[14] Zhang S Zhao L Wang H Efficacy of modified LiuJunZi decoction on functional dyspepsia of spleen-deficiency and qi-stagnation syndrome: a randomized controlled trial. BMC Complement Altern Med 2013;13:54. 23453018 10.1186/1472-6882-13-54 PMC3599864

